# Anti-Cancer Role and Therapeutic Potential of Extracellular Vesicles

**DOI:** 10.3390/cancers13246303

**Published:** 2021-12-15

**Authors:** Naoomi Tominaga

**Affiliations:** Faculty of Clinical Laboratory Science, Yamaguchi University Graduate School of Medicine, 1-1-1 Minami-kogushi, Ube 755-8505, Japan; ntominag@yamaguchi-u.ac.jp

**Keywords:** cancer, extracellular vesicles, exosome, cancer therapy

## Abstract

**Simple Summary:**

Cell–cell communication is an important mechanism in biological processes. Extracellular vesicles (EVs), also referred to as exosomes, microvesicles, and prostasomes, are microvesicles secreted from a variety of cells. Importantly, EVs contribute to cancer malignancy mechanisms such as carcinogenesis, proliferation, angiogenesis, metastasis, and escape from the immune system. As EVs are thought to be secreted into body fluids, they have the potential to serve as diagnostic markers for liquid biopsy. In addition, the characteristics of EVs make them suitable for use in drug delivery systems and novel cancer treatments. In this review, the potential of EVs as anti-cancer therapeutics is discussed.

**Abstract:**

Cell–cell communication is an important mechanism in biological processes. Extracellular vesicles (EVs), also referred to as exosomes, microvesicles, and prostasomes, are microvesicles secreted by a variety of cells. EVs are nanometer-scale vesicles composed of a lipid bilayer and contain biological functional molecules, such as microRNAs (miRNAs), mRNAs, and proteins. In this review, “EVs” is used as a comprehensive term for vesicles that are secreted from cells. EV research has been developing over the last four decades. Many studies have suggested that EVs play a crucial role in cell–cell communication. Importantly, EVs contribute to cancer malignancy mechanisms such as carcinogenesis, proliferation, angiogenesis, metastasis, and escape from the immune system. EVs derived from cancer cells and their microenvironments are diverse, change in nature depending on the condition. As EVs are thought to be secreted into body fluids, they have the potential to serve as diagnostic markers for liquid biopsy. In addition, cells can encapsulate functional molecules in EVs. Hence, the characteristics of EVs make them suitable for use in drug delivery systems and novel cancer treatments. In this review, the potential of EVs as anti-cancer therapeutics is discussed.

## 1. Introduction

Extracellular vesicles (EVs), which also go by exosomes, microvesicles, and prostasomes, are secreted from a variety of cells [1,2] (Table 1). EVs are nanometer-scale vesicles composed of a lipid bilayer and contain biologically functional molecules, such as microRNAs (miRNAs), mRNAs, and proteins [3]. In recent years, EVs have been recognized as a cell–cell communication tool. The basic idea of cell–cell communication using EVs is that EVs secreted from donor cells are taken up by recipient cells in a paracrine or autocrine manner. EVs modify the condition of recipient cells by the biological molecules contained within them. The history of EVs dates back approximately 40 years, when Hans Lutz et al. reported the release of vesicles from old sheep erythrocytes [4]. Ronquist reported a functional fraction in the supernatant of prostatic fluid in the same year [5]. It has been reported that red blood cells secrete vesicles containing proteins and lipids during maturation [6,7,8]. Some research groups have reported that the transferrin receptor is internalized in vesicles made by multivesicular bodies [9,10,11]. However, in 1991, Johnstone et al. concluded that vesicles secreted from cells were “a garbage bin” for unnecessary membrane proteins [12]. Importantly, the vesicles secreted from cells contain mRNA and miRNA, which can be transferred to other cells and be functional in them [13]. Vesicles secreted from cells were removed from the garbage bin and moved into the limelight as a new cell–cell communication tool.

Until the establishment of the International Society of Extracellular Vesicles (ISEV) in 2011, researchers used different names for the vesicles secreted from cells. Hence, to avoid confusion in nomenclature, ISEV encouraged the use of the term “extracellular vesicles (EVs)” for all vesicles secreted from cells [14,15] and offered three other suggestions for nomenclature in their article: (1) State their use of terms explicitly, (2) clearly state their methods, and (3) respect scientific freedom to choose of the nomenclature.

**Figure 1:**
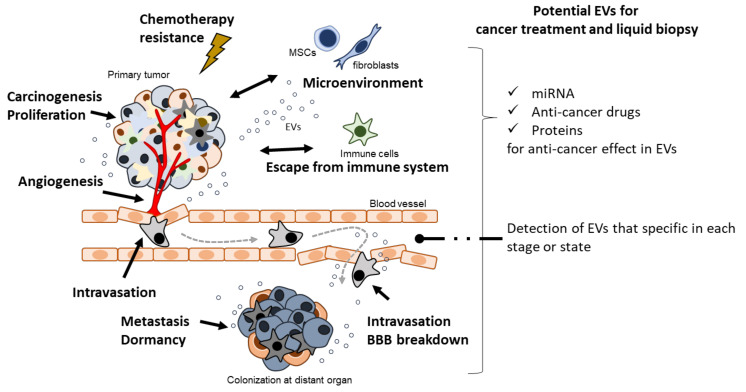
EVs contribute to cancer malignancy and they have an emerging role of therapeutic potential in cancer malignancy.

In the past two decades, EVs have been shown to play a crucial role in cancer biology. Accumulating evidence indicates the importance of cell–cell communication through EVs in cancer malignancy mechanisms, such as cancer cell proliferation [16], immune modulation [17], angiogenesis [18], metastasis [19], and pro-metastasis niche formation [20] (Figure 1). Importantly, in the early history of EV research, EVs derived from dendritic cells (DCs) pulsed with tumor peptides were proposed as a cell-free vaccine method [21]. Furthermore, a potential cancer therapeutic strategy based on the suppression of cancer metastasis via the removal of EVs that contribute to cancer malignancy has been reported [22]. Herein, we summarize and discuss the importance of EVs in cancer biology (summarized in Table 2), as well as their anti-cancer role and therapeutic potential (summarized in Table 3).

## 2. Carcinogenesis

Cancer cells emerge from cells damaged by various factors, such as inflammation, chemical stress, radiation, oxidative stress, and aging [117]. These stresses, especially aging, affect the formation of malignant tumors through the accumulation of genetic and epigenetic changes in genes. It is likely that there is a relationship between these causes of carcinogenesis. Chronic inflammation, such as “inflammaging,” is a risk factor for carcinogenesis, and is caused by cytokines and chemokines [118]. It is predicted that elderly people probably have chronic inflammation without infection caused by senescent cells. The senescence-associated secretory phenotype is a feature of senescent cells that leads to chronic inflammation in elderly people, with factors such as interleukin (IL) -6, and IL-8 secreted by senescent cells [119]. EVs are also secreted from senescent cells, and they may exert detrimental effects [120,121]. Accumulating evidence suggests that EVs contribute to carcinogenesis or precancerous conditions, such as inflammation [26,122,123,124], fibrosis [25,125,126], double-strand breaks in DNA [27,126], and endoplasmic reticulum (ER) stress [127].

Bladder cancer cell-derived EVs induce neoplastic transformation of nonmalignant cells through the induction of the unfolded protein response in the ER [127]. EVs have been suggested to affect tumor recurrence and the potential for carcinogenesis. The Epstein–Barr virus M81-infected B cells release EVs that contain non-coding Epstein–Barr virus-encoded RNA, which are then taken up by B cells [26], which results in chronic inflammation. Inflammation and carcinogenesis caused by viral infection may be linked. EVs derived from macrophages have been reported to upregulate TBC1 domain family member 3 by downregulating stanniocalcin-1-mediated inflammation [122]. A relationship between cholangiocarcinoma and liver fluke infection has been suggested. IL-6 secretion from cholangiocytes is upregulated, and cholangiocytes proliferate after uptake of EVs derived from the liver fluke *Opisthorchis viverrini* [123]. EVs derived from neutrophils, such as miR-23a and miR-155, that infiltrate injured tissue have contributed to double-stranded breaks in DNA [27] increasing inflammation, replication stress, and genomic instability after up taken by surrounding cells. Fibrosis is also known as a precancerous condition [125]. Interestingly, it has been reported that osteosarcoma-derived EVs promote proliferation, migration, adhesion, and sphere formation through MMP-9, TNF-α, IL-6, and transforming growth factor (TGF)-β mRNA expression [128]. EVs may contribute to carcinogenesis by inducing inflammation.

## 3. Proliferation

“Enabling replicative immortality” is a hallmark of cancer [129]. HeLa cells were established in 1953 and contribute greatly to cancer research because of their proliferation on a dish [130]. It is well known that activation of telomerase is one of the causes of proliferation, as it is not subject to the Hayflick limit. Uncontrolled proliferation is a fundamental feature of cancer that leads to gene mutations, metabolic changes, and epigenetic alterations possibly resulting in malignancy. Cancer cell-derived EVs contribute to cell proliferation and growth [131,132,133,134,135].

EVs from mesenchymal stem cells (MSCs) play a dual role in cancer biology. They exhibit potential as anti-cancer treatments but also contribute to cancer malignancy [131,136]. Importantly, MSCs are educated by cancer-derived EVs to contribute to cancer malignancy. It has been reported that EVs derived from cholangiocarcinoma-educated bone marrow MSCs enhance the secretion of C-X-C motif chemokine ligand (CXCL)-1, C-C motif chemokine ligand 2 (CCL2), and IL-6, which affect cancer proliferation [131]. MiR-410 containing EVs derived from human umbilical cord MSCs decreases phosphatase and tensin homolog (PTEN) expression in lung adenocarcinoma. These results suggests that the uptake of EVs by lung adenocarcinoma increases proliferation and decreases apoptosis. Cancer-associated fibroblasts (CAFs) also play a crucial role in cancer proliferation [29]. EV secretion from CAFs increases after gemcitabine (an anticancer drug) treatment and promotes cancer proliferation and drug resistance. Interestingly, it has been reported that EPH receptor A2-enriched EVs from senescent cells promote cancer proliferation [31]. EVs containing miRNAs play important roles in cancer cell proliferation [32].

Long non-coding RNA (LncRNA) is a type of RNA [137]. LncRNAs have many functions, such as the regulation of chromatin states and transcription. TU339, a type of lncRNA, was found in EVs derived from hepatocellular cancer [30]. TU339-containing EVs mediate tumor cell growth and adhesion after transfer to cancer cells. lncRNA-VLDLR also contains EVs that contribute to cellular stress responses [33].

As discussed above, miRNAs, proteins, and lncRNAs in EVs play a critical role in cancer cell proliferation.

## 4. Angiogenesis and Intravasation

Angiogenesis and lymphangiogenesis are important for the survival and progression of cancer cells, which are activated by signals from cancer cells that are growing [138]. These are important steps for the supply of oxygen, nutrients, and metabolism in cancer cells [139]. Vascular endothelial growth factor (VEGF), basic fibroblast growth factor, angiogenin, and TGF-α play an important role in angiogenesis [139]. Cancer-derived EVs also play an important role in angiogenesis [134,140,141,142,143,144,145,146] and lymph-angiogenesis [147]. EVs derived from colorectal cancer cells activate early growth response protein-1 in endothelial cells, causing the migration of endothelial cells [34]. Cancer-derived EVs stimulate MSCs to form vessel-like formations [148]. It has been reported that miRNAs in EVs also plays an important role in angiogenesis [45]. Rac1-, PAK2- [44], VEGF- [46], and angiopoietin-2-containing [48] EVs are related to angiogenesis. These results suggest that cancer-derived EVs promote angiogenesis. Lymph nodes are a route of cancer metastasis [149]. It is reported that laminin γ2-containing EVs promote lymphangiogenesis [50]; however, mechanisms underlying lymphatic intravasation remain unclear.

## 5. Metastasis

Cancer cells can metastasize to any part of the body; however, sites such as bone, the liver, and the lungs are the most common. Brain metastasis is a critical cause of death. Uncontrolled cancer metastasis is a major cause of cancer-related deaths. Cancer metastasis involves multiple steps, such as epithelial–mesenchymal transition (EMT), intravasation, extravasation, and proliferation at the metastatic organ. The seed-and-soil theory is well accepted as a mechanism of metastasis [150]. Using the metastatic efficiency index, Weiss suggested that 65% of metastasis seems to be caused by the amount of organ blood flow [150,151]. In contrast, there are common sites of cancer metastasis and sites that are specific to the cancer type. In such complicated mechanisms of metastasis, EVs contribute to EMT [152,153,154], migration [63,155,156,157], metastasis niche formation [20,158,159], metastasis promotion [158], and the tumor microenvironment [160].

Hypoxia in the tumor environment affects cancer behavior. Secretion of EVs from colorectal cancer cell lines increases under hypoxic conditions [161]. These EVs stimulate the motility, invasiveness, and stemness of colorectal cancer cell lines. Cancer cells communicate with the microenvironment for progression through EVs [61,162,163,164]. MiR-370-3p-containing EVs from breast cancer cells induce IL-6, IL-8, and IL-1β secretion by suppressing the cylindromatosis-/NF-κB-signaling pathway in fibroblasts [58]. It has been suggested that the microenvironment of cancer contributes to cancer progression through education by EVs. EMT is a feature of cancer metastasis in which epithelial cells transition from the epithelial state to the mesenchymal state, and EVs affecting EMT of cancer cells may lead to cancer metastasis [152,153,154]. The migration/invasion step in cancer metastasis is important for migrating to other organs [62,67,68,165,166]. EVs derived from CAFs promote migration and invasion of oral squamous cell carcinoma cells [167]. EVs derived from endothelial cells that are associated with tumors enhance the invasion of cancer cells by changing the cancer microenvironment [156]. The pre-metastatic niche is an idea that distant organs can form a microenvironment that can metastasize cancer cells before they can reach them [20,159,168,169]. EVs derived from cancer cells can change the microenvironment of distant organs and may allow cancer cells to metastasize to distant organs. Brain metastasis is known as a poor prognosis. EVs derived from breast cancer cells break down the blood–brain barrier, making it possible for cancer cells to pass through the biological barrier [19]. MiR-181c plays a role in changing the state of brain blood vessels after being taken up by brain endothelial cells through EVs. It has been reported that tubulin tyrosine ligase-like (TTLL4) is related to EV biogenesis and brain metastasis [57]. Contents of EVs such as miRNAs [63] and lncRNAs [59] play critical roles in cancer invasion and migration.

There is considerable evidence that EVs contribute to metastasis by changing cancer cells and their microenvironment [135,170,171,172,173,174]. EVs derived from breast cancer cells contain small noncoding (nc) RNAs, named orphan ncRNAs, which originate from the 3′ end of the telomerase RNA [60]. Small ncRNAs promote breast cancer metastasis. EVs derived from breast cancer cells upregulate EV secretion via chemotherapy [175]. Drug-induced EVs promote lung metastasis. Interestingly, it has been reported that EVs derived from bovine milk induce cell senescence in cancer cells but promote metastasis by inducing EMT in the primary tumor [176]. In contrast, EVs derived from bone marrow MSCs induce dormancy in metastatic breast cancer cells via miR-23b in EVs [66]. These results indicate that EVs play a critical role in cancer metastasis.

## 6. Escape from Immune System

The immune system consists of macrophages, B cells, T cells, and DCs. It protects the body from invaders such as bacteria, viruses, and toxins; contributes to the recovery of the body; and removes cancer cells. However, cancer cells can escape the immune system by modifying them through EVs. Programmed death ligand-1 (PD-L1) is a receptor that suppresses or stops T-cell reactions by binding to programmed cell death-1 (PD-1). EVs derived from glioblastoma promote immune evasion through PD-1 binding to PD-L1 on EVs [69,70]. Ovarian cancer cell-derived EVs have been reported to inhibit T-cell receptor-dependent activation in T-cells [177]. EVs derived from tumor-associated macrophages have immunosuppressive effects; conversely, these cells have the potential to activate anti-tumor immunity [178]. MSC-derived EVs containing miR-222 contribute to immune escape in colorectal cancer by downregulating the AKT pathway [65]. These reports suggest that EVs derived from cancer cells and cells in the microenvironment suppress the immune system.

## 7. Chemotherapeutic Stress

Cancer chemotherapy has been developed for the treatment of the whole body since 1960s [179], and chemotherapy using cytotoxic drugs has been the main form of therapy for cancer in recent years. Currently, antibodies are used to kill cancer cells directly or via immune cells [180]. Unfortunately, EVs contribute to the evasion of chemotherapeutic agents.

Bone marrow MSC-derived EVs increase the viability of multiple myeloma cells and drug resistance [181]. Non-small-cell lung cancer cell-derived EVs increase gefitinib-induced apoptosis [182]. In EVs, lncRNAs contribute to drug resistance [33,75,76,77,78,79], with lncRNA H19 in EVs increasing gefitinib resistance in non-small-cell lung cancer cells [73]. EVs derived from MSCs increase drug resistance [157]. MSC-EVs are collected under stress using a non-serum culture medium. EVs derived from under-stressed MSCs decrease doxorubicin-induced apoptosis in osteosarcoma cells, and those derived from melanoma cells containing anaplastic lymphoma kinase could transfer drug resistance to other melanoma cells [71]. EVs derived from tumor-associated macrophages, which are components of the cancer microenvironment, increase resistance to the anticancer drug gemcitabine [74]. MiR-30b-3p in EVs derived from hypoxic glioma cells contributes to drug resistance by decreasing the expression of ras homolog family member B [72]. Furthermore, there is a mechanism to resist antibody therapy. Bevacizumab is an antibody used for cancer treatment because of its anti-angiogenic effect, which is discarded through EVs derived from glioblastoma cells after being captured by glioblastoma cells. Interestingly, EVs derived from breast cancer cells showed increased drug resistance in the non-tumorigenic epithelial cell line MCF10A [183]. Cells secrete EVs to resist stress environments, and this ability is acquired by cancer cells.

## 8. Potential of EVs for Liquid Biopsy

EVs secreted into the extracellular environment may be related to mechanisms of cancer malignancy. Many reports have suggested that EVs contain specific molecules that contribute to cancer malignancy or related cancer types. Therefore, it is possible that EVs can be used for diagnosis. A liquid biopsy is a test that uses body fluids such as blood, bone marrow, saliva, urine, and tears. A minimally invasive method is required in liquid biopsy as much as possible to avoid pain. Bone marrow biopsy is an invasive method, as is blood biopsy. On the other hand, saliva, urine, and tears are non-invasive methods of biopsy. Liquid biopsy tests can identify early stage, progression, and metastasis of cancer by detecting specific molecules. The idea of a liquid biopsy using EVs is to detect cancer-specific EV molecules such as miRNAs, DNAs, and proteins for the diagnosis of cancer [184]. As described above, tears have great potential as a non-invasive method in liquid biopsy. EVs in tears can be used for the diagnosis of cancer [185]. However, further evidence is required for its clinical application. Liquid biopsy of prostate cancer uses urine, and is hence a non-invasive method [186,187,188,189,190]. MiR-21, miR-375, and miR-204 have been detected in the urine of prostate cancer patients but not in healthy donors [81]. It has been reported that the levels of miR-221-3p, miR-222-3p, and miR31-5p are higher in high-risk patients compared to low-risk patients [85]. EV proteins are also useful as biomarkers of prostate cancer. FABP5 [80] and androgen receptor splice variant 7 [84] on EVs collected from urine may be used as cancer progression markers. Analyses with blood samples indicate that miR-375, miR-200c-3p, and miR-21-5p are useful for the diagnosis of prostate cancer [87]. There is a combination of EV miRNAs and proteins from the blood [191]. Saliva is a non-invasive bioliquid. EVs collected from saliva may be useful for the diagnosis of lung cancer [192,193,194]. It has been reported that the contents of EVs collected from serum, such as miR-200, lipocalin-2 [86], miR-505-5p [91], ubiquitin C-terminal hydrolase-L1 (UCHL1) [88], cell-free DNA [82,83], and proteins [195,196,197,198,199], are useful for the diagnosis of lung cancer. MiR-193a-5p, and miR-551b-5p have been suggested as biomarkers of malignant pleural mesothelioma. Pancreatic cancer is one of the leading causes of cancer-related mortality [94]. The five-year survival rate of pancreatic cancer is approximately 2%. It is important to detect it at an early stage; however, no diagnostic method for clinical use to identify early-stage tumors or pre-malignant conditions exists. Liquid biopsy using EVs has potential for early diagnosis. Glypican-1 on EVs derived from cancer cells has been found to be a biomarker of early-stage pancreatic cancer [89,90]. Mucins, CFTR, and MDR1 found in EVs from pancreatic juice and cancer cell lines are candidate biomarkers [92,93]. Zinc transporter ZIP4 [37], cytoskeleton-associated protein 4, a novel Dickkopf1 receptor [96], and annexin A1 [200] are also potential biomarkers. A panel of miRNAs, mRNAs, and proteins derived from EVs is useful for the early diagnosis of pancreatic cancer [201,202,203,204,205,206,207]. It may be possible to predict treatment outcomes using EVs [208]. It has also been suggested that miR-133b is a potential biomarker for pancreatic cancer [95]. Interestingly, bacteria-derived EVs collected from blood samples can be used as biomarkers for pancreatic cancer [209]. It has been reported that there are differences in the microbiome between patients with pancreatic cancer and healthy donors and between patients with cancer and the precancerous state in liver disease [210,211]. MiR-150-3p has been suggested as a prognostic biomarker for HCC [97]. MicroRNAs are important diagnostic biomarkers for breast cancer [212,213]. It is important to identify not only miRNAs but also lncRNAs for diagnosis [214]. Thus, evidence suggests that EVs have potential as diagnostic markers for cancer.

## 9. Potential of EVs for Cancer Treatment

As discussed above, EVs from cancer cells and their microenvironment contribute to cancer malignancy. The idea of cancer treatment using EVs has three aspects—(1) inhibition of EV production, (2) disruption of EV uptake, and (3) elimination of EVs [215]. In addition, there is a strategy for the application of EVs that carry a druggable molecule for cancer treatment [216]. CD63 and CD9 are used as markers for targeting EVs in the circulation. EV elimination from the circulation using anti-CD63 and CD9 antibodies can reduce cancer metastasis [22]. The CD9 Fab fragment also inhibits EV internalization [100]. These reports suggest that targeting proteins on EVs can be used for the neutralization and elimination of EVs from the blood. It has also been suggested that potential therapeutics exert anti-cancer effects by blocking EVs containing TGF-β [217]; however, the therapeutics strategy of “inhibition of EV production” is still unclear. Many studies have suggested that EVs can be used as drug delivery systems [218,219,220,221]. EVs derived from a macrophage cell line were loaded with the anticancer drugs paclitaxel or doxorubicin [222] and were then used to inhibit cancer growth. EVs containing hyaluronic acid grafted with 3-(diethylamino)propylamine and the anti-cancer drug doxorubicin can bind to CD44; they exhibit an anti-cancer effect [223]. Organ tropism is required when EVs are used in drug delivery systems. Rabies viral glycoprotein [98], iRGD peptide [224], and integrins [225] have been reported as factors of the organ tropism of EVs. CD47-over express EVs are reduced EV-uptake by macrophages, which can enable the deriver of distant organs [226]. However, the mechanism of organ tropism in EVs remains largely unknown. Interestingly, artificial EVs can be used as drug carriers. An artificial EV was made such that an aptamer carrying bone marrow DCs was extruded from a filter [227]. Numerous reports have shown that EVs containing miRNAs [99,101] exhibit anti-cancer effects. It is a good strategy to load miRNAs that exhibit anti-cancer effects, because EVs containing miRNAs are known. MiR-21 is a critical factor in cancer malignancy [228,229]. EVs containing miR-21 contribute to cancer malignancy and a poor prognosis [159,230]. Anti-cancer effects of miRNAs related to cancer malignancy can be disrupted by EVs containing a miRNA sponge structure. EVs were collected from HEK293T cells expressing the miR-21 sponge construct. These EVs exhibited anti-cancer effects [111]. MSCs are multipotent stem cells used in cell replacement therapy [231]. MSCs have been used to treat multiple diseases to date. Therefore, MSCs are also used as a source of EVs for cancer treatment. MSC-derived EVs containing miRNAs such as miR-206 [112], miR-193a [67], miR-144-3p [113], and miR-125b [114] exhibit anti-cancer effects. MSC-derived EVs contain mi-185 [115]. EVs exert multiple anti-cancer effects through the AKT pathway, which is targeted by miR-185. EVs from adipose-derived MSCs have been suggested to enhance cell apoptosis [232]. In addition, miR-16-5p-loaded EVs inhibited tumor growth in vivo. In contrast, it has been suggested that conditioned media from bone marrow mesenchymal stromal cells also have anti-tumor effects [116]. EVs have been used as a cell-free vaccine for cancer treatment. DCs were cultured with tumor peptides, and then EVs were collected from them [21,102]. EVs derived from tumor-antigen-activated DCs suppressed tumor growth in a T-cell-dependent manner. In addition, it is a good strategy to change the properties of EVs. Depleting CD99 in cells affects the amount of CD99 in EVs, and the effect of EVs can be altered by depleting CD99 in Ewing sarcoma cells [110]. It has been reported that 4T1 cells treated with antibacterial drugs secrete EVs that can inhibit osteoclastogenesis [233]. A natural killer cell line treated with IL-15-secreted EVs can inhibit cancer growth [234]. It is possible that cells treated with reagents secrete EVs that can function as a cancer treatment or inhibit cancer progression. Moreover, EVs from NFAT3-expressed breast cancer cells exert anti-cancer effects [235]. These reports suggest the possibility that EVs derived from specific protein-expressing cancer cells also have anti-cancer effects.

## 10. Conclusions

EVs are microscale vesicles secreted from a variety of cells. EVs contain lipids, proteins, and nucleic acids. EVs participate in cell–cell communication via the molecules they contain. As discussed above, EVs may contribute to cancer malignancy. It is thought that cancer-derived EVs that contribute to cancer malignancy can be detected in body fluids. Many studies have suggested that the detection of EVs may be useful for cancer diagnosis. As described above, EVs are carriers of molecules. Characteristics of EVs indicate their potential for use in drug delivery systems. In addition, many miRNAs exhibiting anti-cancer effects have been identified. EVs seem to be advantageous as carriers of miRNAs because EVs naturally contain miRNAs. Proteins are also naturally contained in EVs. The strategy of “overexpression” of proteins is used to load proteins on EVs. Some reports suggest that it is possible to load anticancer compounds. These methods are important for drug delivery systems. On the other hand, it is necessary to pay attention to EVs because they are a double-edged sword. Considerable evidence suggests that MSCs are a useful source of EVs; however, their mechanisms have also been found to contribute to cancer malignancy in some MSC-derived EVs. Importantly, it has been suggested that standard therapeutics such as anti-cancer drugs can change the nature of cancer-derived EVs from contributing to cancer malignancy to exhibiting an anti-cancer effect. In other words, secreted EVs are heterogeneous and have different contents and proteins. The purification of “druggable” EVs (or removing “wicked” EVs) is important to apply to treatment. Furthermore, the organ specificity of EVs is a key to applying its therapeutics strategy. Transmembrane proteins in EVs responsible for organ tropism are crucial; further elucidation of the mechanisms of organ tropism is required. The mechanisms of EV, such as loading and changing contents in each situation, and the amount of EVs being secreted, are still unknown. Hence, it is necessary to gain deeper understanding of the nature of EVs. In particular, it requires target specificity and the unity of contents of EVs that exclude unnecessary content. Evidence suggests that EVs have great potential for cancer treatment; however, a deeper understanding and research of EVs may be needed for their application to such therapies.

## Figures and Tables

**Table 1:** The features of extracellular vesicles.

	Extracellular Vesicles (EVs)		
Terminology	Exosomes	Microvesicles	Apoptotic Bodies
Origin	Multivesicle body	Plasma membrane	Plasma membrane
Size	50–150 nm	100–1000 nm	100–5000 nm
Marker proteins	CD9, CD63, Tsg101 etc.	Integrins, Selectins, CD40 etc.	Annexin V, thrombospondin, C3b etc.
References	[1,3]	[1,23]	[1,24]

**Table 2:** Cancer malignancy-related EV contents.

	Proteins	miRNAs	lncRNAs	Other
Carcinogenesis		let-7 [25]		EBER2 [26]
		miR-23a, miR-155 [27]		
Proliferation	CLIC1 [28]	miR-410 [29]	TU399 [30]	
	EphA2 [31]	miR-142-3p [32]	lncRNA-VLDLR [33]	
	L1CAM [34]	miR-95 [35]	lncRNA-H19 [36]	
	ZIP4 [37]	miR-30e [38]	EWSAT1 [39]	
		hypoxia-induced miR-155 [40]		
		miR-365 [41]		
		miR-130b-3p [42]		
		miR-497 [43]		
Angiogenesis	Rac1, PAK2 [44]	miR-584-5p [45]		
	VEGF90K [46]	miR-23b, miR-320b [47]		
	angiopoietin-2 [48]	miR-23a [49]		
	laminin γ2 [50]	miR143-3p, miR145-5p [51]		
		miR-141-3p [52]		
		miR-81b-5p [53]		
		miR-4488 [54]		
		miR-10a-5p [55]		
		le-7b-5p [56]		
Metastasis	TTLL4 [57]	miR-370-3p [58]	HLA-F-AS1 [59]	orphan RNA [60]
	PKM2 [61]	miR-181c [19]	HUCL [62]	
		miR-30a-3p [63]		
		miR-185-2p [64]		
		MSC-miR222 [65]		
		miR-30e [38]		
		miR-23b [66]		
		miR-193a [67]		
		miR-622 [62]		
		miR-224-5p [68]		
Escape from immune system	PD-1 [69,70]	miR-222 [65]		
Chemotherapeutic stress	ALK [71]	miR-30b-3p [72]	H19 [73]	
	Vasconcelos, Chitinase 3-like-1 and fibronectin [74]		VLDLR [33,75]	
			HOTPIT [76]	
			RP11-838N2.4 [77]	
			PART1 [78]	
			SNHG14 [79]	

**Table 3:** Liquid biopsy and cancer treatment-related EV contents.

	Proteins	miRNAs	lncRNAs	Other
Liquid biopsy	FABp5 [80]	miR-21, miR-375, miR-204 [81]	-	cell-free DNA [82,83]
	Androgen-receptor splice vairiant 7 [84]	miR-221-3p, miR-222-3p, miR-31-5p [85]		
	Lipocalin-2 [86]	miR-375, miR-200c-3p, miR-21-5p [87]		
	UCHL1 [88]	miR-200 [86]		
	GPC1 [89,90]	miR-505-5p [91]		
	mucins, CFTR, MDR1 [92,93]	miR-193a-5p, miR-551b-5p [94]		
	ZIP4 [37]	miR-133b [95]		
	CKAP4, DKK1 receptor [96]	miR-150-3p [97]		
	Annexin A1 [98]			
Cancer treatment	anti-CD63 antibody, anti-CD9 antibody [22]	miR-134 [99]	-	-
	CD9 Fab fragment [100]	miR-355-5p [101]		
	cell-free vaccine [21,102]	miR-124, miR-128, and miR137 [103]		
		mir-1252-5p [104]		
		miR-320a [105]		
		miR-375 [106]		
		miR-424 [107]		
		miR-203 [108]		
		miR-30a [109]		
		miR199a-3p [110]		
		miR-21-sponge construct [111]		
		miR-206 [112]		
		miR-193a [67]		
		miR-144-3p [113]		
		miR-125b [114]		
		mi-185 [115]		
		miR-16-5p [116]		
